# Reduced inhibitory gate in the barrel cortex of Neuroligin3^R451C^ knock‐in mice, an animal model of autism spectrum disorders

**DOI:** 10.14814/phy2.12077

**Published:** 2014-07-17

**Authors:** Giada Cellot, Enrico Cherubini

**Affiliations:** 1 Department of Neuroscience, Scuola Internazionale Superiore di Studi Avanzati (SISSA), Trieste, 34136, Italy; 2 European Brain Research Institute (EBRI), Rita Levi‐Montalcini Foundation, Rome, 00143, Italy

**Keywords:** Autism spectrum disorders, excitatory/inhibitory balance, Neuroligin3^R451C^ knock‐in mice

## Abstract

Neuroligins are postsynaptic adhesion molecules that interacting with presynaptic neurexins ensure the cross‐talk between pre‐ and postsynaptic specializations. Rare mutations in neurexin–neuroligin genes have been linked to autism spectrum disorders (ASDs). One of these, the R451C mutation of the gene encoding for Neuroligin3 (Nlgn3), has been found in patients with familial forms of ASDs. Animals carrying this mutation (NL3^R451C^ knock‐in mice) exhibit impaired social behaviors, reminiscent of those observed in ASD patients, associated with major alterations in both GABAergic and glutamatergic transmission, which vary among different brain regions and at different developmental stages. Here, pair recordings from parvalbumin‐ (PV) expressing basket cells and spiny neurons were used to study GABAergic synaptic signaling in layer IV barrel cortex of NL3^R451C^ mutant mice. We found that the R451C mutation severely affects the probability of GABA release from PV‐expressing basket cells, responsible for controlling *via* thalamo‐cortical inputs the feed‐forward inhibition. No changes in excitatory inputs to parvalbumin‐positive basket cells or spiny neurons were detected. These data clearly show that primary targets of the NL3 mutation are PV‐expressing basket cells, independently of the brain region where they are localized. Changes in the inhibitory gate of layer IV somatosensory cortex may alter sensory processing in ASD patients leading to misleading sensory representations with difficulties to combine pieces of information into a unified perceptual whole.

## Abstract (precis)

Autism spectrum disorders (ASDs) comprise a heterogeneous group of neurodevelopmental abnormalities, characterized by impaired social interactions. Here, using mice carrying the R451C mutation of the Nlgn3 gene (found in few familial cases of ASDs), and exhibiting behavioral deficits reminiscent of those present in ASD patients, we found that, in layer IV barrel cortex microcircuit, parvalbumin‐expressing basket cells display a reduced probability of GABA release into spiny neurons. Alterations of GABAergic signaling in layer IV somatosensory cortex of NL3R451C knock‐in mice may alter sensory processing leading to misleading sensory representations with difficulties to combine pieces of information into a unified perceptual whole.

## Introduction

Autism spectrum disorders (ASDs) comprise a heterogeneous group of neurodevelopmental disorders characterized by impaired social interactions, deficits in verbal and nonverbal communication associated with stereotyped and repetitive behavior (American Psychiatric Association 2000).

It has been proposed that a disrupted excitatory/inhibitory (E/I) balance in key neuronal circuits accounts for cognitive deficits associated with neurodevelopmental disorders, including ASDs (Gogolla et al. 2009; LeBlanc and Fagiolini 2011; Pizzarelli and Cherubini 2011; Ramamoorthi and Lin 2011; Lin et al. 2013; Zikopoulos and Barbas 2013). At early developmental stages, such disequilibrium may alter synaptic circuits in a period of high plasticity, leading to permanent modifications in neural activity (Zhang and Sun 2011). Interestingly, in the perinatal period, GABA *via* its depolarizing and excitatory action plays a crucial role in shaping and refining neuronal circuits (Cherubini et al. 1991; Ben‐Ari et al. 2007, 2012). This occurs *via* activity‐dependent and ‐independent processes. The latter may involve transmembrane cell adhesion molecules of the neurexin and neuroligin families (Südhof 2008). By bridging the synaptic cleft, these proteins ensure bidirectional signals essential for synapses organization and stabilization (Scheiffele et al. 2000; Varoqueaux et al. 2006; Chubykin et al. 2007; Ko et al. 2009; Poulopoulus et al. 2009).

Several mutations in neurexin–neuroligin genes have been linked to ASDs (Jamain et al. 2003; Laumonnier et al. 2004; Yan et al. 2005; Kim et al. 2008). One of these, the missense mutation R451C of the gene encoding for the postsynaptic adhesion protein Neuroligin3 (*Nlgn3*), found in two families with children affected by ASDs (Jamain et al. 2003), has been introduced by gene targeting in mice (Tabuchi et al. 2007). These mice exhibit major alterations in both GABAergic and glutamatergic transmission, that vary among different brain regions and at different developmental stages (Tabuchi et al. 2007; Etherton et al. 2011; Pizzarelli and Cherubini 2013; Földy et al. 2013). In addition, mutant mice reveal deficits in social interaction similar to those found in ASD patients (Tabuchi et al. 2007). Furthermore, NL3^R451C^ knock‐in (KI) mice exhibit opposite changes in parallel inhibitory systems (Földy et al. 2013), known to play distinct but complementary roles in network oscillations (Klausberger et al. 2005; Bartos et al. 2007). Hence, pair recordings between basket cells, expressing parvalbumin (PV+) or cholecystokinin (CCK), and CA1 pyramidal cells in the hippocampus unveiled a decrease of synaptic efficacy and a loss of tonic CB1 receptor‐dependent suppression of GABA release in PV+ and CCK+ interneurons, respectively (Földy et al. 2013).

Here, we report that in the somatosensory barrel cortex of juvenile NL3^R451C^ KI mice, the impairment of GABA release from PV+ basket cells severely alters the E/I balance in layer IV neuronal microcircuit. This represents a critical issue, since PV+ cells, which are innervated by the same thalamic afferents to excitatory layer IV spiny neurons (Agmon and Connors 1991, 1992; Porter et al. 2001; Sun et al. 2006), play a pivotal role in sensory information, acting as an inhibitory gate for incoming thalamic inputs *via* feed‐forward disynaptic inhibition (Welker et al. 1993; Pinto et al. 2003).

## Materials and Methods

### Ethical approval

All experiments were performed in accordance with the European Community Council Directive of 24 November 1986 (86/609EEC) and were approved by local veterinary authorities and by SISSA ethical committee. All efforts were made to minimize animal suffering and to reduce the number of animal used.

### Animals

NL3^R451C^ KI mice (Tabuchi et al. 2007) were purchased from Jackson Laboratories (Bar Harbor, ME). Experiments were performed on off‐spring males derived from heterozygous mating. Some sets of experiments were performed on double KI off‐spring males obtained by mating heterozygous NL3^R451C^ females with transgenic males expressing enhanced green fluorescent protein (EGFP) in a subpopulation of PV‐containing interneurons (Chattopadhyaya et al. 2004, kindly provided by Dr. A. Bacci, Paris). Electrophysiological experiments were performed and analyzed blind before genotyping. This was carried out on tail biopsy DNA by PCR using a standard protocol. At least five mice for each genotype were used in a given experiment. Control experiments were performed on wild‐type (WT) littermate males.

### Cortical slices

Cortical slices were obtained from postnatal (P) day P9–P14 animals, using a standard protocol (Schubert et al. 2001). Briefly, after being anesthetized with CO_2_, animals were decapitated. The brain was quickly removed from the skull and placed in ice‐cold artificial CSF (ACSF) containing (in mmol/L): NaCl 130, glucose 10, KCl 3.5, NaHPO_4_ 1.2, NaHCO_3_ 25, CaCl_2_ 2, MgCl_2_ 1.3, saturated with 95% O_2_, and 5% CO_2_, pH = 7.3–7.4. Coronal slices (300 *μ*m thick) were cut with a vibratome and stored at room temperature (22–24°C) in a holding bath containing the same solution as above. After incubation for at least 1 h, an individual slice was transferred to a submerged recording chamber and continuously superfused at 33–34°C with oxygenated ACSF at a rate of 2–3 mL/min.

### Electrophysiology

A patch‐clamp amplifier (multiclamp 700b; Axon Instruments, Sunnyvale, CA) was used to record visually identified (with an upright microscope equipped with differential interference contrast optics and infrared video camera) spiny neurons and PV+ basket cells in layer IV of the somatosensory barrel cortex, using the whole‐cell patch‐clamp technique in voltage and current clamp modes. Patch electrodes were pulled from borosilicate glass capillaries (Hingelberg, Malsfeld, Germany); they had a resistance of 5–7 MΩ when filled with an intracellular solution containing (in mmol/L): K‐gluconate 150, HEPES 5, EGTA 1.1, MgCl_2_ 0.5, phosphocreatinine 10. Membrane potential values were corrected for a liquid junction potential of ~16 mV (calculated with the Clampex software; Molecular Devices, Sunnyvale, CA). The stability of the patch was checked by repetitively monitoring the input and series resistance during the experiments. Cells exhibiting >15% changes in either series resistance or holding were excluded from the analysis. The series resistance was <20 MΩ and it was not compensated.

Excitatory postsynaptic currents (EPSCs) and inhibitory postsynaptic currents (IPSCs) were evoked by stimulating afferent fibers through a homemade monopolar silver electrode placed in layer IV or V depending on the experiment. The stimulation frequency was set at 0.1 Hz (stimulus duration 100 *μ*s).

In the experiments in which the integration properties of spiny neurons were measured, inhibitory postsynaptic potentials (IPSPs) were recorded in current clamp mode (after bridge balance correction) using a stimulation intensity as the double respect to the threshold to elicit IPSPs without failures. Artificial (a) excitatory postsynaptic potentials (EPSPs) were simulated by somatic injection of an exponentially rising and falling voltage waveform as:


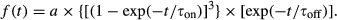
EPSP‐like waveforms had *τ*_on_ and *τ*_off_ of 1.5 and 10 ms, respectively (Fricker and Miles 2000).

For pair recordings, the intracellular solution contained (in mmol/L): KCl 140, MgCl_2_ 1, EGTA 0.5, MgATP 2, HEPES 10. Membrane potential values were corrected for a liquid junction potential of ~4 mV.

### Data analysis

Data were transferred to a computer hard disk after digitization with an A/D converter (Digidata 1322; Molecular Devices). Data acquisition (digitized at 20 kHz and filtered at 3.3 kHz) was performed with pClamp 9.2 software (Molecular Devices). Input resistance (*R*_in_) and cells capacitance were measured online with the membrane test feature of the pClamp software (Molecular Devices, Sunnyvale, CA). Whole‐cell patch‐clamp recordings in current‐clamp mode were used to identify the firing pattern of neurons, in order to distinguish between excitatory spiny neurons (regular spiking) and fast spiking, PV+ interneurons. The adaptation index (AI, Beierlein et al. 2003) was calculated as the ratio between the frequencies of the last and first two action potentials in a train elicited by injecting, at resting membrane potential (*V*_rest_), a long (1600 ms) depolarizing current pulse (100 pA) in recorded neurons.

In voltage clamp experiments, mean EPSCs and IPSCs values were calculated by averaging at least 10 sweeps. The rise time was estimated as the time needed for 10–90% increase of the peak current response. The decay phase was fitted with an exponential function in the form: 
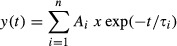


where *τ*_*i*_ and *A*_*i*_ are the time constants and relative fractions of respective components. Synaptic currents were usually fitted with a single exponential. The success rate of unitary IPSCs was calculated as the percentage of successes over 50 trials. The pair pulse ratio (PPR) was measured as the mean peak amplitude of the synaptic response evoked by the second stimulus over that evoked by the first one (the two stimuli were 50 ms apart).

For statistical analysis, the normality of distributions was tested using the SigmaPlot software (Systat Software, San Jose, CA). If not stated differently, statistical significance was tested using unpaired Student's *t*‐test (pClamp software). For data that did not follow a Gaussian distribution, Kolmogorov–Smirnov's test was used. A *P* value < 0.05 was considered statistically significant. Values are given as mean ± SEM.

### Drugs

Drugs were applied in the bath *via* a three‐way tap system, by changing the superfusion solution to one differing only in its content of drug(s). The ratio of flow rate to bath volume ensured complete exchange within 1–2 min. Drugs used were as follows: DL‐AP5, SR 95531 hydrobromide (gabazine), and DNQX purchased from Ascent Scientific (Bristol, UK). Stock solutions were made in distilled water and then aliquoted and frozen at −20°C. DNQX was dissolved in DMSO. The final concentration of DMSO in the bathing solution was 0.1%. At this concentration, DMSO alone did not modify the membrane potential, input resistance, or the firing properties of neurons.

## Results

We first measured, in whole‐cell patch‐clamp experiments (in current and voltage clamp mode), the passive and active membrane properties of layer IV spiny neurons in the somatosensory barrel cortex of WT and NL3^R451C^ KI mice. Comparable values of resting membrane potential (*V*_rest_), membrane capacitance (*C*), and input resistance (*R*_in_) were detected in both genotypes (*V*_rest_: −60 ± 1 and −59 ± 1 mV, *P* = 0.62; *C*: 85 ± 3 and 86 ± 4 pF, *P* = 0.89; *R*_in_: 312 ± 14 and 303 ± 21 MΩ, *P* = 0.71, in WT, *n* = 67, and in NL3^R451C^ KI mice, *n* = 34, respectively). As expected (Feldmeyer et al. 1999), spiny neurons generated trains of adapting spikes in response to long lasting (1600 ms) depolarizing current pulses (100 pA). The firing frequency and the AI were similar in WT and NL3^R451C^ KI mice (the firing frequency was 14 ± 0.7 and 14.4 ± 0.8 Hz, *P* = 0.73; the AI was 0.51 ± 0.03 and 0.54 ± 0.03, *P* = 0.47, in WT, *n* = 67, and in NL3^R451C^ KI mice, *n* = 34, respectively). Taken together, these data indicate that, in both genotypes, recordings were performed from a homogeneous population of neurons, presumably layer IV spiny neurons, with similar passive and active membrane properties.

### Altered E/I balance in layer IV barrel cortex neurons of NL3^R451C^ KI mice

**Figure 1.**
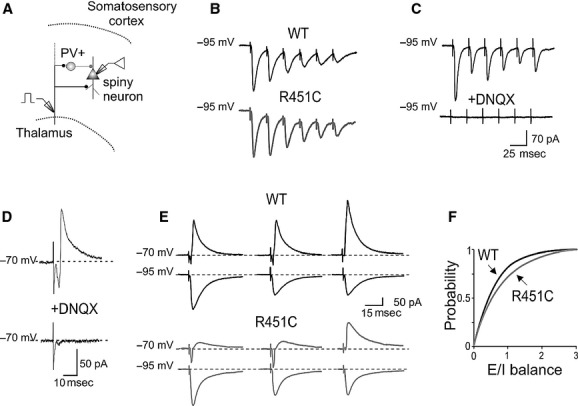
Altered excitatory/inhibitory (E/I) balance in NL3^R451C^ knock‐in (KI) mice. (A) Schematic representation of the microcircuit in layer IV somatosensory cortex showing a PV+ interneuron (circle) and a spiny neuron (triangle). Note that both cells are innervated by the same thalamo‐cortical afferent. Afferent fibers are stimulated through an external electrode placed in layer V, while excitatory postsynaptic current (EPSC) and inhibitory postsynaptic current (IPSC) are recorded from spiny neuron by means of a patch pipette. (B) In both wild‐type (WT; black trace) and NL3 mutant (R451C, grey trace) mice, EPSCs (recorded at −95 mV that corresponds to E_Cl_^−^) were depressed upon stimulation of afferent fibers at 40 Hz. (C) EPSCs evoked in spiny neurons by repetitive stimulation of afferent fibers were blocked by DNQX (20 *μ*mol/L). (D) The EPSC/IPSC sequence was blocked by DNQX (20 *μ*mol/L), indicating that inhibition was disynaptic. (E) Upper traces (on the left) represent EPSCs‐IPSCs sequences evoked by stimulation of afferent fibers (from a holding potential of −70 mV) in spiny neurons from WT (black) and NL3^R451C^ KI mice (grey). Lower traces (on the left) represent isolated EPSCs evoked from the same cells at −95 mV. On the middle, traces digitally scaled to minimize the different driving forces. On the right, isolated EPSCs and IPSCs. IPSCs were obtained by subtracting scaled EPSCs recorded at −95 mV from the EPSCs‐IPSCs sequences recorded at −70 mV. (F) Cumulative probability curves of the E/I balance calculated for each cell by dividing the EPSC for the IPSC amplitudes in WT (black) and in NL3^R451C^ KI mice (grey). The two curves were significantly different (*P *=**0.0003, Kolmogorov–Smirnov's test).

We monitored the amount of excitation and inhibition received by layer IV spiny neurons in response to activation of afferent fibers through a stimulating electrode placed in layer V (see schematic diagram of Fig. 1A). Repetitive stimulation of excitatory inputs at 5–40 Hz produced in both genotypes a robust short‐term depression (STD; Fig. 1B). EPSCs in the train were abolished by the application of DNQX (20 *μ*mol/L), a selective *α*‐amino‐3‐hydroxy‐5‐methyl‐4‐isoxazolepropionic acid (AMPA) receptor antagonist (Fig. 1C). Afferent fibers undergoing STD were activated by single pulses, using a stimulation intensity 1.5‐fold the minimal necessary to evoked an EPSC followed by an IPSC and it was similar in both WT and NL3^R451C^ KI mice (24 ± 6 V in WT, *n* = 24, and 25 ± 9 V, in NL3^R451C^ KI mice, *n* = 13, *P *>**0.88, unpaired *t*‐test).

At −70 mV, between the reversal potential for excitatory and IPSCs (the reversal potential for chloride, E_Cl_^−^, was −95 mV), stimulation of afferent inputs evoked EPSCs followed with similar delays (2.0 ± 0.2 and 2.2 ± 0.2 ms, in WT, *n* = 24, and NL3^R451C^ KI mice, *n* = 13, respectively, *P* = 0.53) by IPSCs. IPSCs were triggered in a feed‐forward disynaptic manner as demonstrated by the brief delays following EPSCs and by their block with DNQX (20 *μ*mol/L; Fig. 1D). At −95 mV, corresponding to E_Cl_^−^, EPSCs were recorded in isolation. Isolated IPSCs were measured after electronically subtracting at −70 mV EPSCs (after appropriate amplitude scaling for the different driving force) from the EPSCs/IPSCs sequences (Fig. 1E). After obtaining for each cell the mean EPSC and IPSC amplitudes, the E/I balance was calculated as the ratio between the peak amplitude of EPSCs and IPSCs. On average, this value was similar in WT and NL3^R451C^ KI mice (0.70 ± 0.11 and 0.98 ± 0.22, in WT and NL3^R451C^ KI mice, respectively). However, it is worth mentioning that in both genotypes the E/I ratios could not be distributed with a Gaussian function. Therefore, cumulative probability curves for this parameter were constructed. With respect to WT animals, in NL3^R451C^ KI mice, the cumulative probability curve was shifted to the right and the difference was statistically significant (Fig. 1F; *P *=**0.0003, Kolmogorov–Smirnov's test). These experiments clearly show that the NL3^R451C^ mutation affects the E/I balance in layer IV somatosensory cortex microcircuit.

### Reduced GABA_A_‐mediated feed‐forward inhibition in NL3^R451C^ KI mice

**Figure 2.**
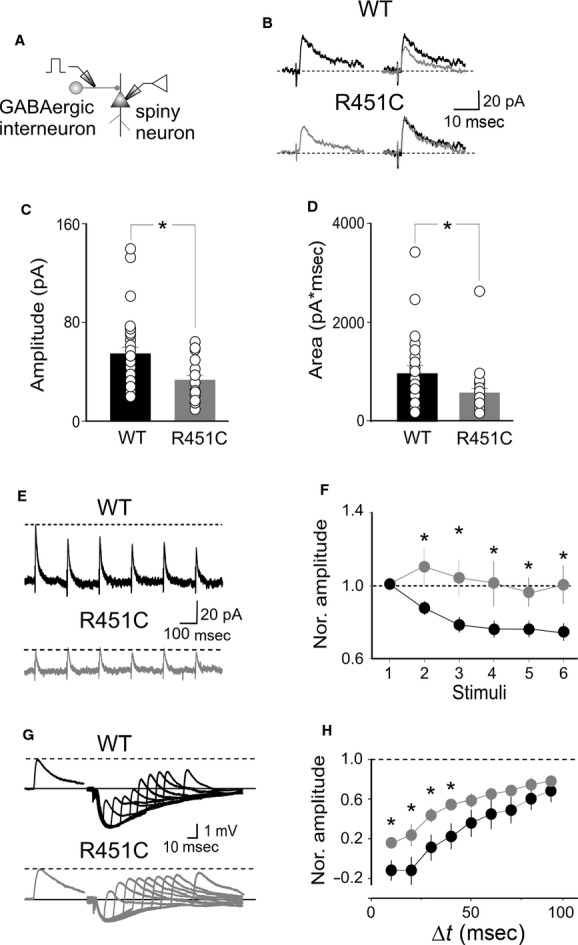
Reduced GABA_A_‐mediated feed‐forward inhibition in NL3^R451C^ knock‐in (KI) mice. (A) Schematic representation of the experimental setting (see text). (B) On the left, representative traces (average of 10) of pharmacologically isolated inhibitory postsynaptic currents (IPSCs) evoked in spiny neurons by stimulation of GABAergic inputs in wild‐type (WT; black) and in NL3^R451C^ KI mice (grey). On the right, IPSCs from the two genotypes are superimposed (upper traces) and normalized (lower traces). (C, D) Each column represents the mean amplitude (C) and area (D) of IPSCs obtained in individual cells (small circles) in WT (black) and NL3^R451C^ KI mice (grey). (E) Sample traces of IPSCs evoked in spiny cells by stimulation of GABAergic inputs at 5 Hz in WT (black) and in NL3^R451C^ KI mice (grey). Note the lack of synaptic depression in NL3^R451C^ KI mice. (F) The amplitudes of consecutive IPSCs normalized to the first ones in WT (black) and in NL3^R451C^ KI mice (grey). (G) Representative traces of a‐excitatory postsynaptic potentials (control traces on the left) injected in the soma of patched neurons at different time after the onset of IPSCs are superimposed in WT (black) and in NL3^R451C^ KI mice (grey). (H) Plot of normalized mean a‐excitatory postsynaptic currents amplitude versus different times (Δ*t*) in WT (black) and in NL3^R451C^ KI mice (grey). In this and in the following Figures vertical bars refer to SEM. *P <**0.05.

The shift to the right of the cumulative probability curve for the E/I ratio observed in NL3^R451C^ KI mice may be caused either by an enhanced excitation or by a reduced inhibition. Since in both WT and NL3^R451C^ KI mice the direct excitatory drive to spiny neurons was not affected (similar EPSCs peak amplitude and latency values were found in both genotypes; EPSCs: 55 ± 13 and 67 ± 19 pA; latencies 2.3 ± 0.3 ms and 2.5 ± 0.3 in WT, *n* = 24, and in NL3^R451C^ KI mice, *n* = 13, respectively; *P *=**0.6 and *P* = 0.5, Student's *t*‐test, for differences in amplitudes and latencies; Fig. 1B), we hypothesized that the E/I unbalance results from a depressed GABA_A_‐mediated feed‐forward inhibition. Therefore, we analyzed pharmacologically isolated IPSCs (with DL‐AP5 20 *μ*mol/L and DNQX 20 *μ*mol/L, to block NMDA and AMPA/kainate receptors, respectively) evoked in principal cells by stimulation of afferents fibers in layer IV–V (Fig. 2A). The stimulation intensity was set as the minimal voltage necessary to evoke postsynaptic responses without failures. As shown in the representative samples of Figure 2B, in comparison with WT animals, IPSCs from NL3^R451C^ KI mice were smaller in amplitude, in the absence of any clear change in their kinetics (see normalized overlapping traces on the right). Overall, the mean peak amplitudes of IPSCs were 54 ± 6 and 31 ± 3 pA in WT (*n* = 46) and NL3^R451C^ KI mice (*n* = 26), respectively (Fig. 2C). This effect was associated with a reduced charge transfer (the areas underlying IPSCs were 976 ± 137 and 568 ± 93 pA*ms in WT and NL3^R451C^ KI mice, respectively (Fig. 2B and D). These values were significantly different (*P *=**0.008 for amplitude and *P* = 0.04 for area, Student's *t*‐test).

Inhibitory postsynaptic currents from WT animals were depressed upon repetitive stimulation of afferent fibers with short trains (six pulses) delivered at 5 Hz (black traces of Fig. 2E and F).

On average (*n* = 46), the amplitudes of consecutive IPSCs, normalized to those of the first ones, were as follows: 0.87 ± 0.03, 0.78 ± 0.03, 0.76 ± 0.04, 0.76 ± 0.04, and 0.74 ± 0.04. In contrast, in NL3^R451C^ KI mice (*n* = 22), IPSCs remained stable during trains (mean values for consecutive normalized IPSCs evoked at 5 Hz frequency were as follows: 1.09 ± 0.10, 1.02 ± 0.11, 1.01 ± 0.12, 0.96 ± 0.07, 1.00 ± 0.10; gray traces of Fig. 2E and F). Differences between WT and KI mice were statistically significant (for the consecutive pulses: *P *=**0.02; *P* = 0.008; *P* = 0.03; *P* = 0.015; *P* = 0.01, Student's *t*‐test).

The inability of GABAergic interneurons from NL3^R451C^ KI mice to undergo frequency‐dependent STD as in littermate controls suggests that a presynaptic dysfunction likely dependent on a reduced probability of GABA release.

To verify how a reduced inhibition integrates incoming excitatory synaptic signals, artificial a‐EPSPs similar in shape to EPSPs evoked by afferent stimulation (~2 mV amplitude) were injected in both WT (*n* = 20) and NL3^R451C^ KI mice (*n* = 22) through the somatic recording electrode at different times (Δ*t*) after the onset of evoked IPSPs (Fig. 2G). Peak amplitudes of a‐EPSPs were measured with respect to IPSPs baseline at different Δ*t* and normalized for the amplitude of a‐EPSPs obtained before IPSP stimulation (Fig. 2H). As shown in the Figure, the amplitudes of a‐EPSPs were larger in NL3^R451C^ KI mice as compared to controls, and a statistically significant difference in the integration window was found between the two genotypes in the interval between 0 and 40 ms from the IPSPs onset (at 40 ms Δ*t*, normalized a‐EPSPs amplitude was 0.22 ± 0.12 ms and 0.55 ± 0.07 in WT and NL3^R451C^ KI mice, respectively, *P *=**0.027 Student's *t*‐test, Fig. 2H). These data indicate that changes in the amount of shunting inhibition during IPSPs account for different integration properties of spiny neurons from NL3^R451C^ KI mice with respect to controls.

### The NL3^R451C^ mutation does not affect the excitatory drive to PV+ interneurons

**Figure 3.**
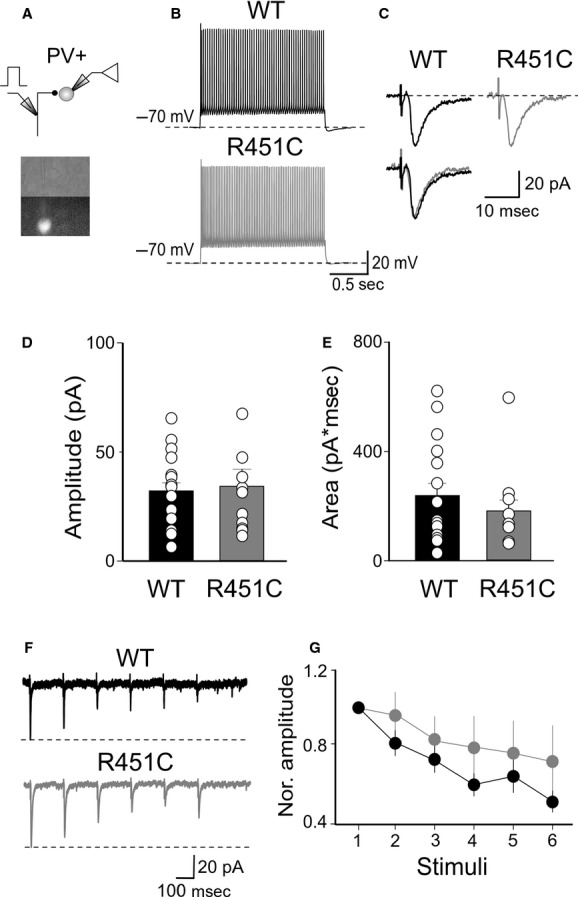
The NL3 R451C mutation does not affect the excitatory drive to PV+ cells. (A) Schematic representation of experimental setting; below, bright field and fluorescent images of the recorded PV+ interneuron. (B) Action potentials firing in response to depolarizing current pulses (100 pA for 1600 ms) in both wild‐type (WT; upper trace) and NL3^R451C^ knock‐in (KI) mice (lower trace). (C) Excitatory postsynaptic current (EPSCs; average of 10, upper traces) evoked in enhanced green fluorescent protein‐positive PV‐expressing basket cells by stimulation of excitatory inputs in WT (black) and NL3^R451C^ KI mice (grey). Below, the two traces are superimposed. (D, E) Each column represents the mean amplitude (D) and area (E) of EPSCs obtained in individual cells (small circles) in WT (black) and NL3^R451C^ KI mice (grey). (F) Sample traces of EPSCs evoked in PV+ cells by stimulation of excitatory inputs at 5 Hz in WT (black) and in NL3^R451C^ KI mice (grey). (G) The amplitudes of consecutive EPSCs normalized to the first ones in WT (black) and in NL3^R451C^ KI mice (grey).

The reduced feed‐forward inhibition observed in spiny neurons following activation of PV+ cells can be attributed to a decreased excitatory input to PV+ interneurons or to a weaker synaptic transmission between PV+ interneurons and principal cells. To distinguish between these two possibilities, recordings were performed from WT and NL3^R451C^ KI mice expressing EGFP in PV+ cells (lower panel of Fig. 3A). These cells exhibited all characteristics of fast spiking interneurons. Their passive membrane properties were comparable in both genotypes (*V*_rest_ −72 ± 2 and −69 ± 2 mV, *P* = 0.36, *C*: 85 ± 7 and 78 ± 7 pF, *P* = 0.5 and *R*_in_: 248 ± 46 and 335 ± 66 MΩ, *P* = 0.28) in WT (*n* = 16) and NL3^R451C^ KI mice (*n* = 12), respectively. In addition, in both genotypes, PV+ cells fired with similar frequency in response to long (1600 ms) 100 pA depolarizing current pulses (32 ± 3 and 31 ± 5 Hz in WT and in NL3^R451C^ KI mice, respectively, *P* = 0.79; Figure 3B (see also Beierlein et al. 2003)).

In the presence of DL‐AP5 (20 *μ*mol/L) and gabazine (10 *μ*mol/L) to block synaptic currents mediated by NMDA and GABA_A_ receptors, respectively, stimulation of afferent fibers in layer V barrel cortex (Fig. 3A) evoked in EGFP‐labeled cells monosynaptic AMPA‐mediated EPSCs with similar characteristics. EPSCs did not show any statistically significant difference in terms of amplitude, charges transfer, and kinetics (amplitude: 31 ± 4 and 34 ± 8 pA, *P* = 0.77; area underlying EPSCs: 238 ± 46 and 185 ± 40 pA*ms, *P* = 0.41; rise time: 1.2 ± 0.2 and 1.5 ± 0.2 ms, *P* = 0.27; decay time: 11.3 ± 2.0 and 8.7 ± 1.1 ms, *P* = 0.28; in WT, *n* = 18, and NL3^R451C^ KI mice, *n* = 15, respectively, Student's *t*‐test; Fig. 3C–E).

In addition, these synapses were highly reliable with almost no failures and exhibited STD (in response to six pulses stimulation delivered at 5 Hz) of comparable amplitude in both genotypes. On average, the normalized amplitude of sixth EPSC over the first one was 0.45 ± 0.07 and 0.68 ± 0.22 in WT (*n* = 18) and in NL3^R451C^ KI mice (*n* = 15), respectively. These values were not statistically significant (*P *=**0.22, Student's *t*‐test; Fig. 3E and F). These data allow excluding that in NL3^R451C^ KI mice the reduced GABAergic signaling to spiny neurons is related to a decreased excitatory drive to PV+ interneurons.

### Reduced probability of GABA release from PV+ cells into spiny neurons in NL3^R451C^ KI mice

**Figure 4.**
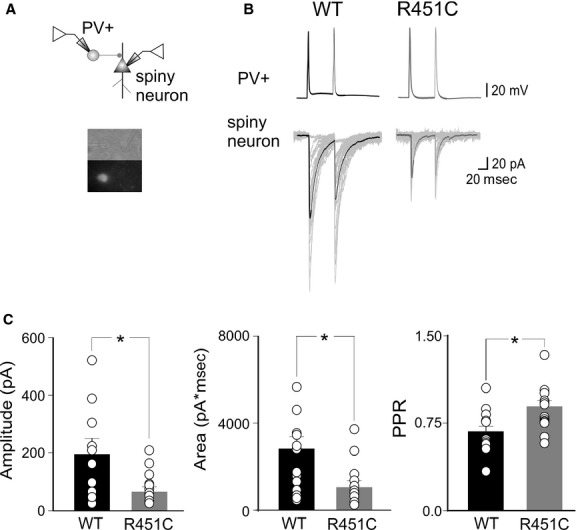
Reduced probability of GABA releases from PV+ cells into spiny neurons. (A) Schematic diagram showing pair recordings from a PV+ cell and a spiny neuron; below, bright field and fluorescent images of the recorded neurons. (B) Representative traces of unitary inhibitory postsynaptic currents (IPSCs) pairs plus failures (the average of 50 individual traces are superimposed) evoked in spiny cells by two action potentials in presynaptic neurons in wild‐type (WT; left) and in NL3^R451C^ knock‐in (KI) mice (right). (C) Each graph represents the mean amplitude (left), area (middle), and pair pulse ratio (right) of unitary IPSCs obtained in individual cells (small circles) in WT (black) and NL3^R451C^ KI mice (grey). *P <**0.05.

To verify whether changes in GABA release from PV+ cells into spiny neurons account for the observed depression of feed‐forward inhibition in NL3^R451C^ KI mice, pair recordings from connected EGFP‐labeled, PV+ interneurons, and spiny neurons were performed (Fig. 4A). Pre‐ and postsynaptic cells were identified on the basis of their respective firing properties. Unitary IPSCs were triggered in spiny neurons by individual spikes, with low failure rate (Beierlein et al. 2003). On average, their peak amplitude was 195 ± 52 and 62 ± 13 pA in WT (*n* = 14) and in NL3^R451C^ KI mice (*n* = 16), respectively, (their respective charges transfers were 2712 ± 652 and 1000 ±259 pA*ms, in WT and NL3^R451C^ KI mice, respectively; Fig. 4B and C). Differences in IPSCs amplitudes and charge transfers were statistically significant (*P* = 0.017 and *P *=**0.018, unpaired *t*‐test). In both the genotypes, IPSCs were completely abolished by the application of 10 *μ*mol/L gabazine (*n* = 3).

Kinetics analysis of unitary IPSCs did not reveal changes between the two different genotypes (for WT, rise time 1.0 ± 0.1 ms and decay time 31.9 ± 1.9 ms; for NL3^R451C^ KI mice, rise time 1.0 ± 0.1 ms and decay time 30.1 ± 2.3; *P *=**0.77 and *P* = 0.55, unpaired *t*‐test). In addition, when unitary IPSCs were triggered by two spikes (50 ms apart), a significantly different PPR was observed between the WT and NL3^R451C^ KI mice (0.66 ± 0.05 and 0.87 ± 0.05, in WT and NL3^R451C^ KI mice, respectively; *P *=**0.011, unpaired *t*‐test). This effect was associated with a slightly reduction, although not statistically significant (*P *=**0.17, unpaired *t*‐test), of success rate (89 ± 4% and 79 ± 5%, in WT and in NL3^R451C^ KI mice, respectively), and in the inversed squared of coefficient of variation (5.4 ± 1.7 and 3.6 ± 1.7 in WT and in NL3^R451C^ KI mice, respectively, *P* = 0.3, unpaired *t*‐test). These results strongly suggest that the impairment of feed‐forward inhibition in NL3^R451C^ KI mice involves a reduced probability of GABA release from PV+ cells into spiny neurons.

## Discussion

The present data unveil an enhanced E/I balance in the microcircuit of layer IV somatosensory barrel cortex of NL3^R451C^ KI mice dependent on the reduced feed‐forward GABA_A_‐mediated inhibition. This controls the temporal window for integrating excitatory inputs thus mimicking in a simple model what could happen in vivo during sensory information processing.

According to Földy et al. (2013), NL3^R451C^ KI mice exhibit “different synaptic phenotypes in distinct brain regions”. Thus, conflicting results have been reported in layer II‐III somatosensory cortex (Tabuchi et al. 2007) and in the hippocampus (Földy et al. 2013; Pizzarelli and Cherubini 2013). In agreement with the results of Földy et al. (2013) obtained from the hippocampus, the present data from the barrel cortex clearly show that a dysfunction of the microcircuit between PV+ interneurons and principal cells may be the unifying event of this particular phenotype, regardless the brain region where it occurs.

Layer V stimulation can orthodromically activate thalamo‐cortical fibers (coming mainly from the ventro‐basal nucleus of the thalamus), fibers coming from layer V pyramidal neuron, and antidromically intracortical branches of cortico‐thalamic fibers (Ferster and Lindström 1985).

We assume that, in our experimental conditions (coronal section of barrel cortex), both EPSCs and IPSCs recorded from spiny neurons in response to the stimulation of afferent fibers in layer V were mainly thalamic in origin since EPSCs experienced a robust frequency‐dependent depression typical of thalamo‐cortical but not cortico‐thalamic inputs (Beierlein et al. 2003). In addition, the contribution of excitatory inputs from layer V to layer IV can be neglected since the connectivity between these two layers has been calculated to be <1% (Lefort et al. 2009). Moreover, it should be mentioned that IPSCs had a disynaptic origin since they were readily blocked by DNQX. The temporal delay of ~2 ms between EPSCs and IPSCs is compatible with a feed‐forward form of inhibition (Agmon and Connors 1992).

We can exclude the involvement of the feed‐back inhibition since the rather low stimulation intensity used (1.5‐fold the minimal necessary to evoke an EPSC/IPSC sequence) was far for reaching the threshold for action potential generation in spiny neurons.

Feed‐forward inhibition is mediated by PV+ cells (Staiger et al. 2009). The R451C mutation of NL3 severely affected the probability of GABA release from PV‐expressing cells. Thus, the reduced amplitude of pharmacologically isolated IPSCs in the absence of any modification of their kinetics, the lack of use‐dependent depression (thought to be the predominant form of short‐term dynamics in many CNS structures, including somatosensory cortex, Tsodyks and Markram 1997), the reduced amplitude of unitary IPSCs triggered in spiny cells by action potentials in EGFP‐positive cells containing PV, an effect associated with increase in PPR, reduction in successes rate and in the inversed square of the coefficient of variation favor a presynaptic type of action. Interestingly, in accord with the present data, a reduced probability of GABA release was detected also at synapses formed by PV+ basket cells onto CA1 pyramidal neurons in the hippocampus (Földy et al. 2013), indicating, as already mentioned, that the primary targets of the NL3 mutation are PV‐expressing basket cells, independently of the brain region where they are localized (see also Gogolla et al. 2009).

How can the NL3 mutation affect synaptic strength at PV+ basket‐principal cell synapses? NL3 is highly expressed in the brain where, unlike other postsynaptic adhesion molecules of the NLs family, is located at both glutamatergic and GABAergic synapses (Budreck and Scheiffele 2007; Levinson et al. 2010; Baudouin et al. 2012). Although the functional role of this adhesion molecule is still poorly understood, the R451C mutation leads to almost 90% retention of the protein in the endoplasmatic reticulum (Tabuchi et al. 2007), a condition that reduces the binding activity of the remaining molecules to *β*‐neurexin 1 (Kleijer et al. 2014). This may modify the transynaptic signaling of these molecules whose interaction with other partners remains largely unknown. Compatible with the multitude of effects of the R451C mutation on neurotransmission, as suggested by Földy et al. (2013), NLs may participate in the balance array of diverse functions possible *via* their interactions with multiple ligands.

In cortical neurons, precisely time‐locked responses are triggered by somatosensory stimuli (Phillips et al. 1988; Arabzadeh et al. 2005). Such temporal accuracy is essential for sensory representation and it is under the control of thalamo‐cortical feed‐forward inhibition (Gabernet et al. 2005). Therefore, alteration of the inhibitory gate in layer IV somatosensory cortex of NL3^R451C^ KI mice may affect sensory processing in ASD patients leading to altered sensory representations with difficulties to combine pieces of information into a unified perceptual whole (Jasmin et al. 2009; Orekhova et al. 2012; Paton et al. 2012; Stevenson et al. 2014). In addition, the enhanced cortical excitability, resulting from the reduced feed‐forward inhibition, may profoundly impact on cortical states known to regulate many aspects of behavior (Poulet et al. 2012).

Whatever is the mechanism, the shift in the E/I balance observed here may destabilize synaptic circuits and may alter the generation of gamma rhythms (Brunel and Wang 2003; Mann and Mody 2008; Wright 2009), believed to be the neural correlates of high cognitive functions (Basar et al. 1999; Wang 2010). Rhythmic oscillations at gamma frequency strongly rely on the activity of fast‐spiking parvalbumin‐positive cells that have been proposed to represent reference signals for temporal coding and sensory binding in large neuronal ensembles (Bartos et al. 2007; Cardin et al. 2009; Sohal et al. 2009). Disruption of gamma rhythms may account for cognitive deficits found in ASD patients (Orekhova et al. 2007; Gross et al. 2012; Richard et al. 2013; Snijders et al. 2013).

## Acknowledgment

We are grateful to Majid Moshtagh‐Khorasani for technical assistance in designing the a‐EPSP.

## Conflict of Interest

None.
